# Pleasure-Inclusive Sex Education, Sexual Agency, and Sexual Well-Being in Adolescents and Young Adults: A Scoping Review

**DOI:** 10.1007/s10508-025-03103-8

**Published:** 2025-03-10

**Authors:** Jenneke van Ditzhuijzen, Amely Overeem

**Affiliations:** 1 Interdisciplinary Social Science, Social Policy and Public Health, Utrecht University, Padualaan 14, 3584 CH Utrecht, The Netherlands; 2 Gynecology and Obstetrics and Amsterdam Reproduction and Development, Amsterdam University Medical Center, Amsterdam, The Netherlands; 3 Seksueel Welzijn Nederland, Utrecht, The Netherlands

**Keywords:** Sexual pleasure, Sex education, Sexual agency, Sexual well-being, Scoping review

## Abstract

It is known that incorporating pleasure into sex education can promote condom use (e.g., Zaneva et al., 2022), but it remains unclear whether this approach also contributes to sexual agency and sexual well-being more broadly. Pleasure-inclusive sex education is designed to enhance sexual agency, which, in turn, may facilitate sexual well-being. In this study, we review the literature on two key topics: (1) the associations between pleasure-inclusive sex education and sexual agency, and (2) the links between sexual agency and sexual well-being. We conducted a search across five scientific databases using a broad range of variables related to pleasure-inclusive sex education, sexual agency, and various aspects of sexual well-being, focusing on research from the last two decades. A total of 33 articles were selected for inclusion. In Part 1 of the review, we found that all studies reported positive associations between pleasure-based sex education and sexual agency or sexual well-being. However, the literature was marked by limitations, preventing definitive conclusions about the added benefits of the pleasure component. In Part 2, we found positive associations between sexual agency (and related variables) and sexual well-being. While sexual agency appears to be linked to increased sexual well-being, it remains unclear whether pleasure-inclusive sex education plays a significant role in this relationship. Given these findings, there is a strong need for high-quality research using innovative evaluation designs that consider other important sources of sex education and contextual factors.

## Introduction

Exploring relationships and sexuality is a key and normative aspect of growing up (Delamater & Friedrich, 2002; Tolman & McClelland, 2011), with adolescence serving as a critical transition period in this process. Sex education plays a crucial role in helping young people develop the competencies and sexual agency necessary for healthy sexuality and positive sexual well-being (Chandra-Mouli et al., 2015; Kågesten & Van Reeuwijk, 2021). Integrating pleasure into sexuality education not only reduces sexual health risks (Zaneva et al., 2022) but may also enhance sexual agency by empowering youth with the knowledge and skills to pursue their desires and refuse unwanted experiences. In turn, this empowerment could contribute to broader sexual well-being.

Even though the pursuit of sexual pleasure is a primary motivation for engaging in sexual activity (Laan et al., 2021; Meston & Buss, 2007; Starrs et al., 2018), it is often overlooked in sex education. In the Netherlands, where 96% of high schools include sex education in their curriculum, only 22% incorporate sexual pleasure (Rutgers & DUO, 2022). However, there is growing interest in positive, comprehensive, and pleasure-based approaches, which are associated with better sexual health outcomes (Kohler et al., 2008; Landers & Kapadia, 2020). The effects of integrating pleasure into sex education on sexual well-being remain unclear, as do the associations frequently cited as key components of the mechanisms underlying these potential effects. The current study aims to review and synthesize the literature on the relationships between pleasure-inclusive sex education, sexual agency, and sexual well-being.

### The Concept of Sexual Well-Being

Many studies on sexual well-being begin with the World Health Organization’s (2019) definition of sexual health, rather than well-being. This definition of sexual health encompasses not only the absence of disease and coercion but also pleasurable sexual experiences. In contrast, the concept of “sexual well-being” remains less clearly defined, with no consensus on its conceptualization (Lorimer et al., 2019; Sundgren et al., 2022). In fact, a review found that only 10 out of 162 studies measuring sexual well-being provided a definition (Lorimer et al., 2019). Moreover, sexual well-being is frequently conflated with sexual health, and distinguishing the two is challenging due to their strong interrelationship. This lack of conceptual clarity may contribute to confusion and hinder the use of sexual well-being as a valid outcome in public health interventions (Mitchell et al., 2021).

The lack of a universal definition of sexual well-being presents challenges for researchers seeking to measure it. To address this issue, some researchers employ multiple measures of sexual well-being, while others develop their own measures. Measures of sexual well-being vary widely, with some assessing aspects of one’s sex life, others focusing on satisfaction judgments, and still others including factors such as satisfaction with sexual relationships, sexual awareness, sexual self-esteem, and body image esteem (for an overview, see Sundgren et al., 2022). Some researchers have defined sexual well-being as the comparison between an individual’s current and ideal sex life (Byers & Rehman, 2014). Lastly, sexual well-being has been conceptualized as “sexual well-being freedom,” which refers to a person’s ability to achieve sexual well-being, or their real opportunities and liberties (Lorimer et al., 2019). In this sense, sexual well-being is not only individually experienced but is also influenced by social and structural factors.

In defining sexual well-being, we draw from Mitchell et al. (2021), who proposed a model in which sexual well-being is firmly related to sexual pleasure, sexual justice, and sexual health, forming four overlapping pillars. Their suggested domain of sexual well-being includes sexual safety and security (including absence of sexual violence or victimization), sexual respect, sexual self-esteem, resilience in relation to sexual experiences, forgiveness of past sexual experiences, self-determination in one’s sexual life, and comfort with sexuality. For the current study, we included a wide variety of operationalizations of sexual well-being, to fully capture the literature using outcomes of sexual well-being. To prevent overlap with Zaneva et al. (2022), we excluded variables related to sexual health in the narrower sense, such as risk on STI/HIV.

### The Concept of Sexual Agency in Relation to Sexual Well-Being

Sexual agency has typically been understood as the ability to initiate sex, make sexual choices, and communicate one’s sexual wishes and boundaries (Vanwesenbeeck et al., 2021). Definitions often focus on autonomy, and sexual agency has been operationalized as sexual assertiveness, sexual self-efficacy, and related capacities. These capacities are very different conceptually, but clearly related to each other, and they are often measured using instruments with items that have a similar content. Even though these concepts are often conceptualized as individual capacities, scholars also emphasize that the navigation of sexuality takes place within a social and structural context, such as the context of family and peer relations, and the cultural and legal context that individuals live in, which strongly shapes this agency (e.g., Cense, 2018a; Vanwesenbeeck et al., 2021). In line with this, scholars have argued that sexual agency should be conceptualized as ubiquitous, instead of an outward performance of an internal attribute. This way we can move from changing potential victims to changing pervasive, systemic threats to sexual well-being (Bay-Cheng, 2019).

Kågesten and van Reeuwijk (2021) use theories and literature to formulate a competency-based framework, which increases adolescents’ agency. In the context of this framework, sexual agency forms the link between competencies and sexual well-being. Sexual agency may increase the potential for pleasurable and positive sexual experiences or other outcomes of sexual well-being. Associations between sexual communication, self-esteem, and efficacy on the one hand and sexual well-being on the other have indeed been found (Mastro & Zimmer-Gembeck, 2015), yet it is still unclear to what extent sexual agency, or related concepts such as sexual autonomy, have an impact on sexual well-being. We are aware that different conceptualizations are not interchangeable, but for readability reasons, we will use the term sexual agency as an umbrella term to include sexual autonomy, assertiveness, self-efficacy, and other related concepts.

### Pleasure-Inclusive Approaches to Sex Education

The pleasure approach to sex education is not a new one. Over three decades ago, feminist scholar Fine (1988) called for a genuine discourse of desire, which would invite adolescents to explore what feels good and bad, desirable and undesirable, grounded in experiences, needs, and limits. This discourse would release females from a position of receptivity and enable an analysis of the dialectics of victimization and pleasure. However, only in the last decade or so, this approach is gaining traction again in research. This may have to do with the current focus on the health benefits of incorporating pleasure in sex education, as opposed to a strictly gender equality-based framework.

Most sex education programs still focus on reducing risks, and evaluations generally do not take pleasure outcomes into account (Lameiras-Fernández et al., 2021). However, there are several potential benefits of adding a pleasure-inclusive approach into sex education. First, reviews have found that there is evidence that a pleasure-based approach to condom use (“putting the sexy into safe sex”) increases condom use and contributes to the prevention of STI/HIV as well as unintended pregnancies (Hanbury & Eastham, 2015; Scott-Sheldon et al., 2015; Zaneva et al., 2022). The systematic review and meta-analysis of Zaneva et al. (2022) found an overall moderate positive effect on condom use, but also that pleasure-incorporating interventions can have positive effects on attitudes toward safe sex. However, adding sexual pleasure to sex education may also have a more fundamental effect on sexual well-being because it increases the potential for positive sexual experiences for all (Laan et al., 2021; Mark et al., 2021), enhances social emotional learning, and may also decrease the potential for negative sexual experiences. To our knowledge, a review of the evidence on this latter association, the one between pleasure-inclusive sex education and sexual well-being as distinct from sexual health outcomes like STI/HIV risk, is still lacking.

### Associations Between Pleasure-Inclusive Sex Education, Sexual Agency, and Sexual Well-Being

Empowering young people to make healthy choices, and to be agentic, autonomous, or assertive about their boundaries, is often a large component of sex education programs (Cense, 2018a, 2018b). The idea is that encouraging young people to take responsibility for their sexuality will prevent adverse outcomes such as STIs or sexual violence. At the same time, being agentic, assertive, and autonomous may also increase sexual well-being more broadly, because it provides the tools for taking up a position and embodying a sexual self within their own social and cultural context (Cense, 2018b).

Looking at sexual health in its narrowest sense, pleasure-inclusive sex education may prevent STIs and unintended pregnancy “because it seeks to empower young people—especially girls and other marginalized young people—to see themselves and others as equal members in their relationships, able to protect their own health” (Haberland & Rogow, 2015, p. S15). Applying this to the concept of sexual well-being, pleasure-inclusive sex education may reinforce sexual agency by empowering people to situate oneself and one’s choices in a social context (Cense, 2018a), but also to make or influence decisions and assert own interests (Kågesten & van Reeuwijk, 2021). The next step is that this increased sexual agency increases the potential for improved sexual well-being, which also includes the freedom to achieve this sexual well-being (Lorimer et al., 2019) without experiencing or perpetrating sexual violence.

**Fig. 1:**
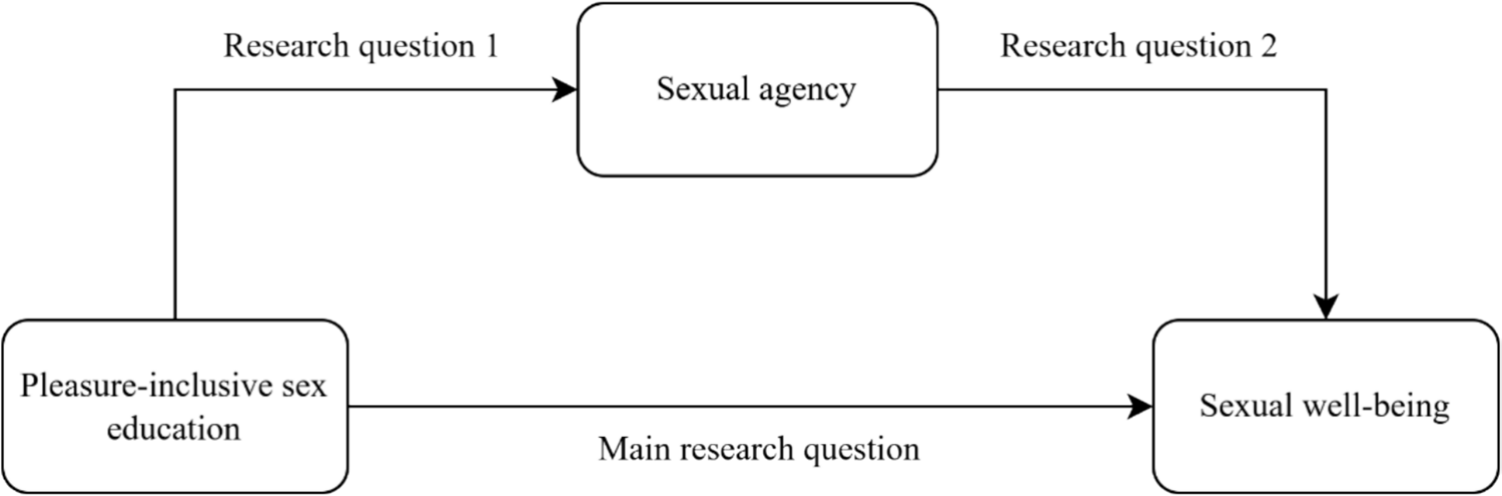
Conceptual model

To inform and guide science, policy, and practice, a thorough and critical review of the literature is needed. The current study aims to fill this gap, by reviewing the literature on associations between pleasure-incorporating sex education, sexual agency or related concepts, and sexual well-being. We did this by looking for studies on associations between pleasure-inclusive sex education or sexual health interventions and sexual well-being (main Research Question), between pleasure-inclusive sex education and sexual agency (Research Question 1), and between sexual agency and sexual well-being (Research Question 2). Figure 1 illustrates the relationships studied in this scoping review. The model is suggestive of a mediation, but since no mediation study like this has been performed, the current study gathers the available evidence on each of the arrows in the model.

## Method

### Study Design and Preregistration

The research questions were proposed by Rutgers, a Dutch expertise center on sexuality and a developer of a substantial part of the sexuality education programs used in the Netherlands, in co-creation with Seksueel Welzijn Nederland. The authors developed the protocol and search strategy. The protocol was preregistered at OSF (osf.io/v3wrz). The review is a scoping review in the sense that we explored the full range of the literature on the variables of interest, but for Research Question 1 and the main Research Question we also used a systematic approach and used Cochrane effectiveness evaluation, where this was possible.

### Search Strategy

As a starting point for our search strategy, we used previously conducted literature reviews as an example. Based on these reviews, we composed an overview of used terminology in similar research. These terms were aligned with the main and sub-questions of our research. Some terms were omitted to avoid overlap with the literature review of Zaneva et al. (2022). Zaneva et al. focused on articles about pleasure-based intervention in relation to safe sex and condom and contraceptive use. Therefore, those terms were not included in this review. We searched using a wide scope of variables related to pleasure-inclusive sex education (e.g., pleasure-based/sex-positive sex education/school intervention), sexual agency (e.g., sexual autonomy, assertiveness, or self-esteem), and sexual well-being (from sexual satisfaction to sexual revictimization risk), see Table 1 for the final terms. These final terms were combined into search strings, specified per database. We searched in five databases (CINAHL, PsycINFO, PubMed, Scopus, and Web of Science) for relevant literature in the last two decades, between January 1, 2002 and March 6, 2023. In addition, we made use of a previously performed non-systematic literature review (Overeem & Van Ditzhuijzen, 2022), from which some relevant articles were extracted. After executing the search strategy, all found articles were exported to EndNote 20 to remove duplicates. Subsequently, the remaining articles were exported to Rayyan, where the articles were screened and assessed.

**Table 1:** Terminology search strategy per variable

Positive sex education
Pleasure-based OR pleasure-inclusive OR sex-positive* OR positive OR sexual pleasure OR pleasure OR comprehensive OR rights-based AND Sex* education OR sex* and relationship education OR relationship and sex* education OR sex* health promotion OR sex* health education OR sex* training OR sex* program* OR sex* school intervention OR sex* information OR reproductive education OR reproductive health education OR erotic education
*Autonomy*
Autonomy OR agency OR self-efficacy OR self-determination OR self-esteem OR self-love OR empowerment OR assertiveness OR decision ownership OR confidence OR consent OR sexual rights awareness OR sexual expression
*Sexual well-being (including prevention of sexual violence/(re-)victimization)*
Sexual well-being OR healthy sexuality OR sexual enjoyment OR healthy relationships OR relationship quality OR relationship satisfaction OR sexual pleasure OR sexual violence OR sexual victimization OR sexual assault OR sexual abuse OR sexual harassment OR sexual intimidation OR nonconsensual penetration OR unwanted sexual contact OR rape OR sexual safety OR sexual happiness OR sexual respect OR sexual satisfaction OR orgasm frequency

### Screening and Selection

The selection procedure was performed in steps, in line with the Preferred Reporting Items for Scoping Reviews (PRISMA-ScR; Tricco et al., 2018). Due to the large volume of articles, the screening was divided between the two researchers. Eighty-nine percent was screened by AO and 11 percent was screened by JvD. To guarantee interrater reliability, we calibrated the screening and consulted each other regularly, and discussed the inclusion of studies thoroughly.

**Fig. 2:**
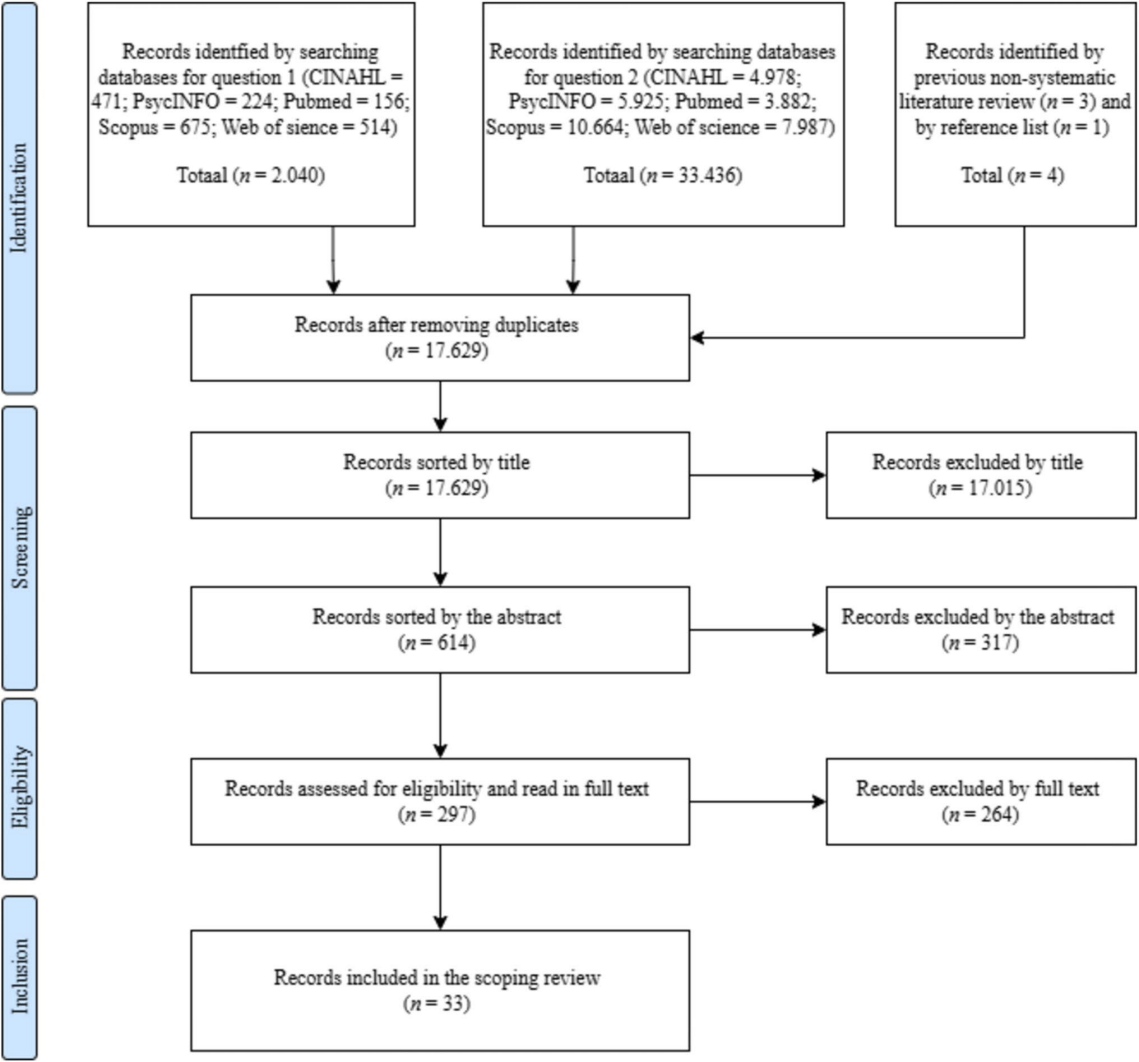
PRISMA flowchart

The identified articles were first selected or excluded based on their title. All articles that were not about the subject of the main or sub-questions were excluded. In this phase, rejection criteria were: not in English language, no variable(s) of interest, focus on risk behavior/no sexual pleasure component, or a focus on condom use. For example, we also excluded studies that focused on general assertiveness, rather than sexual assertiveness. The remaining articles were then screened based on the abstract. We checked if the articles focused on the right target group (adolescents and young adults between ± 12 until ± 30 years old), if the research was original (e.g., not a review study), and if the association between the variables was tested in the desired direction (e.g., the effect of sexual agency on sexual well-being and not the other way around). After that, the remaining articles were read in full text and assessed for their eligibility. Lastly, we removed articles that were not specific for sexuality, e.g., about general assertiveness and prevention of revictimization. All remaining 33 articles were included in this scoping literature review (see Fig. 2).

### Knowledge Synthesis

For data analysis, we reviewed all included studies using a standard format in Excel. Categories assessed were study design, sample size, age group, variables, measurements, reliability of measurements, modes of reporting, and outcomes, as well as which Research Questions were answered.

### Quality Evaluation Strategy

Of the studies related to the main Research Question and Research Question 1, only four could be assessed using a standard Cochrane format (RoB 2), because they made use of a waitlist controlled design. These studies made use of control groups of people who were on the waiting list for the intervention, but had not taken part in it yet. The other studies addressing Research Question 1 used either a pre-intervention—post-intervention comparison (without control group), or cross-sectional retrospective design. The quality of the studies addressing Research Question 2 was assessed based on predefined quality assessment criteria related to bias, strengths, and limitations: (1) information/measurement bias, response bias, and selection bias; (2) study design, sample size, representativeness of participant group; and (3) inclusion of control- and confounding variables. For calibration, both authors independently assessed the quality of 14 studies, after which assessments were discussed and agreed upon. Of the rest of the records, 76% was rated by AO and 24% by JvD.

## Results

The results are presented in two sections. The first section describes the literature related to Research Question 1 and the main Research Question. The second section describes the literature related to Research Question 2. After this, we describe methodological challenges in general, zoom in on the strongest studies, and describe the main findings of this scoping review.

### Part 1: The Impact of Pleasure-Inclusive Sex Education on Sexual Agency and Well-Being

For Research Question 1, the selection resulted in eight cases. These intervention studies indicated that, following the intervention, participants demonstrated increased knowledge of relationships (Ashworth & Carton, 2022), sexuality, and pleasure preferences, as well as greater self-confidence and more positive attitudes (Ashworth & Carton, 2022; Raymond & Hutchison, 2019). Additionally, participants showed enhanced sexual agency or assertiveness (Levin et al., 2012; Raymond & Hutchison, 2019), improved self-defense self-efficacy (Raymond, 2019; Raymond & Hutchison, 2019), greater sexual-body esteem, and higher self-efficacy in experiencing sexual pleasure/orgasms (Guitelman et al., 2019). They also reported feeling more comfortable discussing sex (Hensel et al., 2021), thinking less in terms of rape myths and the sexual double standard (Levin et al., 2012; Raymond, 2019), and exhibiting increased confidence in managing conflicts (Webermann et al., 2022). However, most studies were characterized by methodological challenges.

An important issue was the lack of clarity regarding whether the intervention actually included information on sexual pleasure or was simply categorized as “positive” (Ashworth & Carton, 2022; Crocker et al., 2019; Levin et al., 2012; Webermann et al., 2022). In cases where pleasure was included, it remained unclear to what extent pleasure was emphasized in the intervention, if it had a large or a smaller role (Raymond, 2019; Raymond & Hutchison, 2019). Only two studies explicitly focused on pleasure-based interventions; however, these were programs specifically and solely designed to enhance pleasure, rather than pleasure-focused sexuality education aimed at improving sexual well-being.

The first study on an intervention that was clearly pleasure-based was Guitelman et al. (2019). Participants were instructed to read the book “Becoming cliterate” of Laurie Mintz. The study found that the book was effective in enhancing sexual functioning, but they found only small magnitude pretest to posttest changes in sexual assertiveness, compared to the waitlist control group. According to Guitelman et al. (2019), this sex-positive intervention has helped young women become more comfortable with their own bodies and their own genitals, more assertive about their sexual needs, and more confident in achieving sexual pleasure. The second study investigating a pleasure-based intervention was Hensel et al. (2021). They focused on effects of the OMGyes online resource, and found that participants’ knowledge about their pleasure preferences increased pretest to posttest, as well as their confidence and positive sexual experiences. This study did not have a control group. These studies are interesting, but focus on pleasure mostly, and do not provide information about pleasure-based comprehensive sexuality education or more general outcomes related to sexual well-being. We also did not find any RCT or experimental design studies comparing a pleasure-inclusive program with a non-pleasure-inclusive program, or the same program with and without pleasure component.

We closely examined the four waitlist control studies that could be assessed using the Cochrane framework (Guitelman et al., 2019; Raymond, 2019; Raymond & Hutchison, 2019; Webermann et al., 2022; see Table 2). All four studies were conducted in the USA, using convenience samples of college students from universities, with a mean age ranging from 20 to 23.5 years. Webermann et al. (2022) was the only study to include both male and female participants, and it was also the sole study to focus on the perpetration of sexual violence as an outcome. The other three studies had all-female samples. According to the Cochrane evaluation, only Webermann et al. (2022) demonstrated a low concern for risk of bias, while the remaining studies showed varying levels of concern, from moderate to high (see Table 2). However, it is important to note that the intervention in Webermann et al. (2022) focused on positive relationship communication rather than sexual pleasure. All in all, the quantitative evidence does not clearly support a causal relationship between pleasure-based sexuality education and sexual agency or sexual well-being.

**Table 2:** Characteristics of included studies on the association between pleasure-inclusive sexuality education and sexual agency (Research Question 1) or sexual well-being (main Research Question)

Authors (year)	Sample and country	Study design	Pleasure-inclusive sexuality education	Sexual agency related variables	Sexual well-being variables	Important findings	Methodological characteristics
Ashworth and Carton (2022)	14 people with intellectual disabilities in the UK	Pre–post comparison within group experimental design	Let’s talk about: sex intervention; positive sexual health education for individuals within secure ID settings	Sexual knowledge, attitudes, confidence		Results showed improvement across all sub-factors of the SAK (sexual attitudes and knowledge scale), but no statistically significant change	Low N, specific group, unclear to what extent intervention is pleasure-based
Crocker et al. (2019)	13 stakeholders (e.g., teachers, parents, youth) in Australia	Qualitative semi-structured interviews	Program positive adolescent sexual health conference	Building confidence, skills, resilience, knowledge	Sexual health and well-being	Stakeholders perceived that young people were engaged to strengthen their sexual health and well-being due to many factors, which followed three themes: a safe and open learning environment, empowerment of young people, and involvement of the support system and broader community	Hard to compare to others due to design. Participants were already involved in the topic (likely to see positive aspects). The intervention focuses on positive sexual health but it is unclear if any component is actually pleasure-based
Guitelman et al. (2019)	50 college students (age 18–26) in the USA	Pretest–posttest randomized waitlist control group design, survey	Bibliotherapy intervention. The book examined was “Becoming Cliterate” (Mintz, 2017) which combines feminist analysis and self-help for women’s orgasm difficulties, empowering women to orgasm and to continue their own sex-positive education	Entitlement to pleasure from self, from partner, efficacy in achieving pleasure, sexual self-reflection, body esteem, self-sexual assertiveness	Orgasm rates, satisfaction with orgasm, overall sexual satisfaction, attitudes toward women’s genitals, and overall sexual functioning	In the intervention group, small to large pretest to posttest effect sizes were found for two measures of orgasm, attitudes toward women’s genitals, sexual-body esteem, self-efficacy in achieving sexual pleasure, arousal, sexual satisfaction, sexual pain, sexual assertiveness, and overall sexual functioning; a small effect size for one of the two orgasm measures was found in the control group	Waiting list comparison group. Overlap in intervention variable and outcome. Cochrane evaluation: Some concerns. Favors experimental outcome direction
Hensel et al. (2021)	870 adult women (of which 183 were under 25) in the USA	Pretest–posttest within group experimental design	OMGyes, online educational resource, presents research-informed strategies for women’s pleasure	Sexual agency, sexual knowledge, confidence and positivity, sexual pleasure, self-knowledge	Perceived impact on sexual attitudes, sexual pleasure experiences	Statistically significant, large effect size increases in participants’ knowledge about their own pleasure preferences, their confidence and positivity about that knowledge, as well as how pleasurable their sexual experiences were during both masturbation and partner sex	Reliability/validity analysis is missing. Questions were asked in one direction only. Participants were website users, so they were already motivated to improve their sexual pleasure
Levin et al. (2012)	332 undergraduate students (age 17–22) in the USA	Cross-sectional survey, convenience sample	Retrospective reflection on sex ed by parents and peers: Positive-sexuality (versus sexual double standard or abstinence) subscale (reflecting the discourse that sex is positive, natural, and egalitarian)	Sexual agency, comfort in sexual communication, sexual self-efficacy	Rape myth acceptance, unwanted sexual experiences, victimization, perpetration	Positive-sexuality messages were positively associated with sexual agency/communication, sexual self-efficacy and (negatively) with rape myth acceptance.	Sex ed measured retrospectively. Relationships can be bidirectional/reversed. Sex ed was regarded “positive” but unclear to what extent it was pleasure-based
Raymond and Hutchison (2019)	63 female university students in the USA	Pilot test, waitlist-comparison group design, quasi-experimental	RSVP program (sex-positive sexual violence prevention) focusing more broadly on healthy relationship skill development	Sexual assertiveness, attributions of blame, sexual communication; self-defense self-efficacy	Positive sexual self-understanding	Treatment group improved in self-defense self-efficacy, thematic analysis reported behavioral and attitudinal changes; qualitative responses noted changes in their behavior related to areas such as sexual communication, self-blame, and assertiveness	Pilot study. Only one of five variables showed differences between intervention and control group in terms of pretest–posttest difference, the others did not. It is unclear whether participants knew they were in the waitlist group. The waitlist group had already started the intervention at Time 2; participation was not blinded, and this assessment did not compare pleasure inclusive with pleasure exclusive variant. Cochrane evaluation: Some concerns. Unpredictable outcome direction
Raymond (2019)	173 female university students (mean age = 20.8) in the USA	Quasi-experimental (nonrandom assign to control/exposure), pre- and posttest	RSVP program (sex-positive sexual violence prevention) focusing more broadly on healthy relationship skill development	Sexual communication, willingness to intervene against sexual aggression, self-defense self-efficacy	Rape myth acceptance, sexual double standards, positive sexual self-understanding	Decrease of rape myth acceptance, increase of self-defense efficacy	No random assignment, indirect measures of sexual well-being (intentions). Cochrane evaluation: High/some concern. Favors experimental/unpredictable outcome direction
Webermann et al. (2022)	62 college students, 55% female (mean age = 23.5) in the USA	Experimental design, random assignment to group/waitlist, pre–post–post testing	Skills for Healthy Adult Relationships (SHARe), weekly eight-session (12-h) group program for college students	Enhancing positive communication, decreasing negative communication, building interpersonal skills such as conflict management promoting positive relationship attitudes, (c) strengthening the ability to self-regulate in relationships	Perpetration of IPV, perpetration of emotional abuse	SHARe participants reported significantly higher confidence in their ability to manage conflicts at post-intervention and significantly lower psychological aggression at the follow-up compared with waitlisted controls. At the three-month follow-up, self-reported perpetration of psychological abuse was 1.5 times higher for waitlist controls versus SHARe participants	Small sample size, limited statistical power; intervention is aimed at positive communication in relationships, but not specifically pleasure-based. Cochrane evaluation: Low concern. Favors experimental outcome direction

There were also some studies using qualitative and mixed methods designs. Raymond and Hutchison (2019) used sequential explanatory analysis in their mixed methods study, i.e., they used qualitative findings to explain their quantitative results by answering the question “how did attending the RSVP intervention impact the participants?”. The thematic analysis of the answers to open-ended questions resulted in themes such as increased awareness or increased knowledge, and feeling “stronger in telling people what I want and how I want it.” Crocker et al. (2019) evaluated stakeholder perceptions of the Australian Positive Adolescent Sexual Health Conference, a sexual health promotion intervention incorporating parents, peer educators, community workers, and teachers. They found that young people were engaged to improve their sexual health through a safe and open learning environment, involvement of the social environment, and lastly, empowerment of young people. Both studies suggest that agency does play a role in improving sexual well-being in interventions.

### Part 2: Associations Between Sexual Agency and Sexual Well-Being (Research Question 2)

In this review, we found 25 studies investigating linkages between sexual agency and sexual well-being (Table 3). These studies did not investigate the impact of pleasure-inclusive sex education. Of these studies, 15 focused on positive sexual experiences and 11 on negative sexual experiences as outcomes. Most studies used a cross-sectional design (n = 19). Only five studies were based on longitudinal studies, but of these, one used data from one wave only, and was therefore considered cross-sectional. In addition, we found one qualitative study.

**Table 3:** Characteristics of included studies on the association between sexual agency and sexual well-being (Research Question 2)

Authors (year)	Sample and country	Study design	Sexual agency related variables	Sexual well-being variables	Important findings	Methodological characteristics
Alarcão et al. (2022)	260 Cape Verdean immigrant and Portuguese native women and men in Portugal	Cross-sectional telephone interviewing survey	Sexual autonomy, sexual distress	Sexual pleasure	Sexual autonomy was positively associated with sexual pleasure among Cape Verdean and Portuguese women and Portuguese men. Sexual distress was negatively associated with sexual pleasure among women, especially Cape Verdean women who reported higher sexual distress	Small sample, cross-sectional data, low reliability of sexual autonomy scale
Berg et al. (2022)	2037 Undergraduate students (mean age = 20.5) in the USA	Cross-sectional survey	Sexual assertiveness, social anxiety	Sexual victimization	The indirect effect of social anxiety on sexual victimization via sexual assertiveness was significant for all five types of sexual victimization	Cross-sectional data. Relationship can be bidirectional/reversed
Blunt-Vinti et al. (2018)	30 Student women (age 18–25) in the USA	Qualitative semi-structured interviews	Sexual agency and societal acceptance of women’s sexuality	Sexual satisfaction	Analyses showed three dominant themes: communication with sexual partners, sexual self-awareness and acceptance, and sources of information and education. All three themes fit broadly under women’s sexual agency and societal acceptance of women’s sexuality	Explorative study
Chmielewski et al. (2020)	348 emerging adults (age 18–26) in the USA	Cross-sectional survey	Embodiment, sexual desire, entitlement to sexual pleasure, sexual agency	Sexual pleasure	Women’s positive connections to their bodies were associated with greater comfort with their sexual desire, which in turn was associated with both greater entitlement to sexual pleasure and sexual agency in the service of pleasure and protection	Cross-sectional data. Relationship can be bidirectional/reversed. Unclear if they controlled for confounding
Grose (2017)	430 Undergraduate women (mean age = 19.9) in the USA	Cross-sectional survey, convenience sample	Sexual empowerment/critical consciousness about gender (mediated by sexual subjectivity and assertiveness)	Sexual pleasure	Support was found for sexual empowerment processes in which critical consciousness about gender was indirectly related to sexual pleasure through relationships with two mediators, sexual subjectivity and sexual assertiveness	Cross-sectional data. Relationship can be bidirectional/reversed, extensive study with many variables
Higgins et al. (2011)	2168 University students (mean age = 20.1) in the USA	Cross-sectional survey	Self-esteem, sexual self-concept, sexual guilt, sexual self-comfort	Physiological sexual satisfaction, psychological sexual satisfaction	In multivariate analyses, significant (*p* < .05) correlates of both physiological and psychological satisfaction included sexual guilt, sexual self-comfort, self-esteem (especially among men), relationship status, and sexual frequency	Cross-sectional data. Relationship can be bidirectional/reversed. Unclear how variables are measured
Impett and Tolman (2006)	116 Girls in 12th grade (age 16–19) in the USA	Longitudinal, but only 1 wave used, so cross-sectional	Sexual self-concept, approach sexual motives	Sexual experiences, sexual satisfaction	Both sexual self-concept and approach sexual motives were associated with greater sexual experience across a broad range of sexual behaviors. Furthermore, sexual self-concept and approach sex motives predicted higher sexual satisfaction at most recent intercourse	Cross-sectional data. Relationship can be bidirectional/reversed
Jozkowski (2013)	640 College students (age 18–27) in the USA	Cross-sectional survey	Consent (internal and external)	Quality of intercourse	For women, three internal consent factors explained a significant proportion of variance in quality of intercourse. For men, one internal and two external consent factors were significant predictors	Cross-sectional data. Relationship can be bidirectional/reversed. Retrospective design
Katz et al. (2010)	87 female undergraduates (mean age = 18.0) in the USA	Longitudinal survey	Self-blame, sexual refusal assertiveness	Sexual (re)victimization	Those who reported initial sexual victimization at Time 1 were more likely than other women to report later college victimization at Time 2. Path analyses showed that self-blame and decreased sexual refusal assertiveness (SRA) explained this effect	Mediators are measured at baseline at the same time as predictor variable, so it’s hard to say what came first. No confounder control and no control for background demographics
Kelley et al. (2016)	296 college women (age 18–25) in the USA	Longitudinal assessments	Prior sexual victimization (mediated by sexual assertiveness and resistance self-efficacy)	Sexual victimization	Results of structural equation modeling indicated that the relationship between baseline and follow-up sexual assault was mediated by the study variables. Follow-up analyses suggested that sexual assertiveness served as a mediator	Mediators are measured at baseline at the same time as predictor variable, so it’s hard to say what came first
López-Alvardo et al. (2022)	538 emerging adults who have had a committed relationship in Ecuador	Cross-sectional survey	Sexual assertiveness	General well-being and relationship satisfaction	In women, sexual assertiveness was associated with general mental well-being and relationship satisfaction; but not in men	Cross-sectional data. Self-selected homogenous sample, not representative
Ménard and Offman (2009)	71 men and women (mean age 27.6) in Canada	Cross-sectional survey	Sexual self-esteem (mediated by sexual assertiveness)	Sexual satisfaction	The results showed strong correlations between all three variables and confirmed sexual assertiveness as a partial mediator of the relationship between sexual self-esteem and sexual satisfaction	Cross-sectional data. Relationship can be bidirectional/reversed. No control for confounding
Miller et al. (2016)	2893 adolescents (age 12–18) in South-Africa	Longitudinal survey	Sexual situation self-efficacy	Forced sexual experiences	Youth are more likely to experience forced sex after periods of time when their levels of self-efficacy are lower. Furthermore, youth who are lower in self-efficacy compared to their peers are more likely to experience forced sex	Outcome variable measured with only one item
Oattes and Offman (2007)	74 adults (mean age = 27.4) in Canada	Cross-sectional survey	Sexual self-esteem	Sexual communication	Analyses of the responses of 74 individuals indicated that high levels of both global and sexual self-esteem predicted a higher ability to communicate about satisfying sexual behaviors with a partner. Hierarchical regressions demonstrated that sexual self-esteem was a unique predictor of sexual communication over and above the contribution of global self-esteem	Cross-sectional data. Relationship can be bidirectional/reversed. Unclear if they controlled for confounding
Oesterle et al. (2022)	673 college women in the USA	Cross-sectional survey	Sexual refusal assertiveness, assertive resistance strategy intentions	Sexual assault severity	High sexual assault refusal assertiveness and endorsement of assertive resistance strategy intentions may serve as protective factors against reassault among women who enter college with a more severe sexual assault history	Cross-sectional data using retrospective questions. Limited confounder control. Convenience college sample
Peixoto et al. (2018)	438 students (mean age = 22.4) in Portugal	Cross-sectional survey	Sexual functioning mediated by sexual self-esteem	Sexual satisfaction	Sexual self-esteem appears to partially mediate the relationship between sexual functioning and sexual satisfaction	Cross-sectional data. Relationship can be bidirectional/reversed. Unclear if they controlled for confounding
Perkins et al. (2023)	406 black women (mean age = 27) in the USA	Cross-sectional survey	Sexual assertiveness	Sexual satisfaction	SWS dimensions moderated the association between sexual assertiveness and sexual satisfaction. Findings from the present study highlight the importance of considering culturally salient racialized gender schemas when examining Black women’s sexual attitudes and behaviors	Cross-sectional data. No confounder control
Phillips (2014)	353 sorority and non-sorority women (97% < 23) in the USA	Cross-sectional survey	Sexual assertiveness questionnaire	Sexual victimization (sexual experiences)	Sexual assertiveness and refusal assertiveness were negatively correlated with sexual victimization	Cross-sectional data. Relationship can be bidirectional/reversed. Retrospective design
Schry and White (2013)	672 college women (mean age = 19.4) in the USA	Cross-sectional survey	Social anxiety mediated by sexual refusal assertiveness	Sexual victimization	Social interaction anxiety was significantly positively related to likelihood of experiencing coerced sexual intercourse, and significant indirect effects, via decreased sexual refusal assertiveness, were found for both coerced sexual intercourse and rape	Cross-sectional data. Relationship can be bidirectional/reversed. Retrospective design
Townsend et al. (2020)	284 undergraduate psychology students (mean age = 20.0) in the USA	Cross-sectional survey	autonomous motives and nonautonomous motives for sex	Victimization, negative well-being	Autonomous motives were positively associated with sexual victimization in women but not in men. Compared to autonomous motives, sex for nonautonomous motives was linked to less self-esteem in both sexes, and with more depression and sexual victimization in women	Cross-sectional data. Relationship can be bidirectional/reversed. Men are underrepresented
Van Bruggen et al. (2006)	402 university women in Canada	Cross-sectional survey	Sexual self-esteem	Sexual revictimization	Structural equation modeling indicated that the relationship between child abuse (i.e., CSA and child psychological maltreatment) and sexual revictimization was partially mediated by sexual self-esteem, sexual concerns, and high-risk sexual behaviors	Cross-sectional data. Relationship can be bidirectional/reversed. Retrospective design
Verbeek et al. (2020)	248 sexually experienced adolescents (mean age = 14.7) in the Netherlands	Longitudinal cohort study	Sexual autonomy	Positive and negative emotions after having sex	Adolescents’ sexual autonomy appears to play a particularly important role in how they experience having sex. Parent–adolescent sexual communication also related to sexual autonomy, yet no indirect effects	High quality study, but relatively low (sub)samples
Walker et al. (2011)	335 college women (age 17–24) in the USA	Cross-sectional survey	Relational sexual assertiveness	Verbal sexual coercion and rape	Findings suggest that sexual assertiveness is related to fewer experiences of sexual coercion	Cross-sectional data. Relationship can be bidirectional/reversed. Unclear if they controlled for confounding
Wongsomboon et al. (2022)	401 women (mean age = 23.9) in the USA	Cross-sectional survey	Sexual assertiveness as a mediator	Orgasmic function in casual sex	Greater pleasure (autonomous) motives related to higher sexual assertiveness, which in turn related to higher orgasmic function in casual sex	Cross-sectional data. Relationship can be bidirectional/reversed. Self-selection bias according to researchers
Zimmer-Gembeck et al (2015)	363 unmarried women (age 18–25) in Australia	Cross-sectional survey	Sexual self-efficacy, entitlement to desire	Satisfaction, emotional reactions to most recent sexual encounters	As expected, young women were more satisfied and reported more positive emotional reactions and fewer negative reactions to their most recent sexual encounters, when they felt more entitled to desire and reported greater sexual self-efficacy	Cross-sectional data. Relationship can be bidirectional/reversed. Retrospective design

The cross-sectional studies found significant associations between sexual agency (or related concepts) and sexual well-being, but the strength and scope of these effects varied strongly. We took a closer look at the longitudinal studies using multiple timepoints (Katz et al., 2010; Kelley et al., 2016; Miller et al., 2016; Verbeek et al., 2020). Katz et al. (2010) and Kelley et al. (2016) found that sexual assertiveness could largely explain revictimization and mediated the relationship between first and later victimization, but both studies measured the mediator variable at baseline at the same time as the predictor variable. Therefore, it is hard to say what came first: low sexual refusal assertiveness or having experienced sexual (re)victimization. Miller et al. (2016) found that young people were more likely to be victims of sexual violence when their level of self-efficacy was lower than normal, and lower compared to peers. Verbeek et al. (2020) found in the Dutch “Project Stars” that when adolescents scored higher on sexual autonomy or global self-esteem at a young age, they experienced more positive sexual emotions later in life. Despite its smaller sample size, this study is the strongest in our selection for the second Research Question.

## Discussion

In this review, we examined the existing literature on the associations between pleasure-inclusive sex education, sexual agency (or autonomy, assertiveness), and sexual well-being. We deliberately took a broad approach to explore what is currently known about these associations, while systematically assessing the quality of the studies using standard critical appraisal criteria whenever possible.

Most studies found positive associations between pleasure-based sex education and sexual agency and/or sexual well-being. However, many of these studies were limited in their ability to assess causality. While all studies reported outcomes in the hypothesized direction, it remains unclear whether the pleasure component itself was responsible for the observed changes, or whether the intervention’s broader focus on sexuality was the key factor. Therefore, we conclude that there is insufficient evidence to support a causal relationship between pleasure-based sex education and sexual agency or sexual well-being.

There is stronger evidence that pleasure-based interventions improve sexual health in the narrow sense, like STI/HIV risk, as described in Zaneva et al. (2022). Sexual well-being is much less frequently evaluated compared to STI/HIV risk in public health research (Bond & Ford, 2024). This may be why there is more robust evidence for those outcomes in relation to pleasure-based sexuality education, compared to sexual well-being or sexual agency. However, the fact that potential benefits of these interventions are difficult to substantiate, does not imply that the interventions are not helpful or beneficial. Some studies do show that these interventions may be promising in increasing sexual well-being. Therefore, high-quality evaluation studies are still sorely needed.

All studies that investigated linkages between sexual agency and sexual well-being found various significant positive associations, but most of the studies had a cross-sectional design. There were, however, some longitudinal studies of relatively good quality. Based on these, we can conclude, with caution, that sexual agency variables may indeed have the potential to increase the likelihood of positive sexual experiences and decrease the likelihood of negative sexual experiences.

### Methodological Challenges of the Reviewed Literature

A large part of the studies had a cross-sectional design, which does not exclude the possibility of reversed and/or bidirectional associations. Sex-positive messages, for example, can increase sexual agency, but people who are more sexually agentic may also be more open to hearing sex-positive messages. A frequent problem with pretest posttest intervention studies is that attitudes and knowledge can rapidly change based on personal experiences, natural development, social desirability, or external stimulants. Moreover, lack of a control group means that found effects can be related to the mere exposure to any (sexuality) intervention on the topic; not specifically the pleasure-inclusive part of it. Also, overestimation of effect just after the intervention is common and nearly all studies in this review were based on self-report. In addition, participants were not always randomly sampled or divided between waitlist and intervention group (e.g., Higgins et al., 2011; Raymond, 2019). The longitudinal design may be stronger in terms of establishing the sequence of events and therefore more apt for investigating causal questions, yet it is no golden panacea either. Samples are often smaller, more often from the same group (e.g., a particular school), and participants are often lost to follow up; oftentimes this attrition is, to some extent, selective. Sampling problems occurred when convenience samples of participants were recruited without concern for the representation of the population, for example, students enrolled in psychology or health courses, or women who attended a healthcare clinic. Even though it is difficult to avoid self-report, the problem remains that sexual victimization and perpetration are typically underreported whereas sexual well-being measures can be biased in both directions, depending on the context and the question.

In sum, the field is characterized by various methodological limitations, which complicates conclusion formation. These limitations may have to do with challenges that are inherent to investigating this sensitive topic in current societies, but also with the fact that many studies are exploratory or pilot studies.

### Limitations of This Study

This scoping review resulted in an overview of the evidence about associations between pleasure-based sex education, sexual agency, and sexual well-being. Despite a systematic approach, this review may not have yielded the full range of evidence. It is possible that we have missed some effective interventions because the study did not indicate “pleasure” or “positive” or similar variables as part of the intervention.

Another limitation is of a more conceptual nature. This review investigated literature on associations between three variables, but none of these studies investigated the mediating or explanatory role of sexual agency (or related variables) in the relationship between pleasure-inclusive sex education and sexual well-being. A focus on agency as the only explanatory mechanism, may put unwarranted responsibility on potential victims of sexual violence, and it is unclear how sexual agency and perpetration may be related. Conceptualizing this link as the theory of change for prevention of victimization, is, in essence, a form of victim blaming, a harmful narrative contributing to victim stigmatization (Cherniawsky & Morrison, 2022). Also, pleasure-inclusive sex education can have an impact on sexual well-being through, for instance, increased knowledge on how sexual pleasure can be achieved. Therefore, sexual agency cannot and should not be seen as “the only way” in which pleasure-inclusive sex education may affect sexual well-being.

Furthermore, the conceptualization of pleasure-inclusive sex education was somewhat fuzzy, which may have led to the exclusion of relevant research. In this review, we did not assess the actual program content or the extent to which the interventions were genuinely pleasure-based; however, we observed significant variability in this regard. The level of pleasure-inclusiveness in most of the included studies can be questioned, and those that were clearly pleasure-based had a limited scope in terms of outcomes related to sexual well-being. To advance this field and draw conclusions about the impact of pleasure-based sex education on sexual well-being, it is essential to identify the core elements and define what constitutes a pleasure-inclusive intervention. For example, the mere message that "pleasure is important" is insufficient, as real-world sexual encounters are not always immediately pleasurable. Instead, they involve a learning process of understanding what feels good, what does not, and how to connect with one’s sexual partner(s). Clumsy and uncertain first experiences are also part of this process, provided they are consensual.

In the current study, we focused on sexual agency, and not so much on consent. Incorporating pleasure into sex education can foster communication around safe and pleasurable experience (Koepsel, 2016). Furthermore, pleasure-inclusive sexuality education may be impactful not only for its content, but also for the style of the intervention, particularly in providing a safe space for exploring topics around sex and relationships. Since consent is a core concept in the promotion of sexual well-being, future research should also include this topic. The relationship between consent and pleasure is no yet clear, and needs further investigation. Nevertheless, this review provided an extensive overview of existing literature from the past 20 years, and identified evidence gaps as a starting point for new research.

### Implications

This review shows that RCTs evaluating the effect of sex-positive interventions on sexual well-being are lacking. However, the focus on the RCT as a “golden standard” has been criticized (e.g., Hein & Weeland, 2019). In theory, it should be possible to use a randomized controlled design to compare a group getting a pleasure-inclusive sex education program with a group that receives the same program without the pleasure component, and a group receiving no sex education at all. In practice, however, this is costly and highly challenging to realize, and it may very well be possible that laboratory-found results cannot be replicated in real-world settings. Investigating long-term effects of pleasure-inclusive sex education is even more challenging, as it may be extremely difficult if not impossible to isolate effects of this sex education from potential other sources of sex education, such as parents and peers, as well as confounding factors that may affect sexual well-being. Strong and sound research is needed to advance the field. Yet, some methodological issues may be inherent to these types of research questions and not (easily) solvable. It is therefore too early to answer the question if pleasure-inclusive sex education “works” to improve sexual well-being and/or sexual agency. To advance the field, a closer dialogue between researchers and practitioners is needed (McGeeney & Kehily, 2016) to evaluate the effects of core “active ingredients” of pleasure-inclusive sex education. Further, alternative more reflexive, context-appreciative evaluation methods may be a way forward that is more fruitful and informative for policy and practice.

The fact that we found limited evidence for a causal relation between pleasure-inclusive sex education and sexual agency or sexual well-being, does not mean that it is absent or that a sex-positive approach does not have any positive impact. There is compelling evidence that sexual pleasure improves sexual health, mental health, and physical health (e.g., Anderson, 2013; Klein et al., 2022; for a review, see also Laan et al., 2021). Additionally, a focus on pleasure may also lead to more gender equality. Currently, not all individuals have equal opportunities for pleasurable sexual experiences (Laan et al., 2021; Van Lunsen et al., 2013). The call for pleasure in sex education, as stated by Fine in 1988, continues to be relevant: to learn about pleasure rather than procreation helps in focusing on similarities rather than differences, and prioritizing pleasurable sex without the deprioritization of pleasure of sexual partners, may shift norms to more gender equal ones (Laan et al., 2021).

## Data Availability

Not applicable.

## References

[CR1] Alarcão, V., Stefanovska-Petkovska, M., Candeias, P., & Pascoal, P. M. (2022). Exploring intersectional variations in sexual pleasure, sexual autonomy, and important correlates. *Social Sciences,**11*(11), 496. 10.3390/socsci11110496

[CR2] Anderson, R. (2013). Positive sexuality and its impact on overall well-being. *Bundesgesundheitsblatt, Gesundheitsforschung, Gesundheitsschutz,**56*(2), 208–214. 10.1007/s00103-012-1607-z 23361205 10.1007/s00103-012-1607-z

[CR3] Ashworth, S., & Carton, H. (2022). “Let’s talk about: Sex”: Development, pilot and evaluation of a positive sexual-health education group for individuals within secure ID settings. *Journal of Intellectual Disabilities and Offending Behaviour,**13*(1), 1–11. 10.1108/JIDOB-03-2021-0005

[CR4] Bay-Cheng, L. Y. (2019). Agency is everywhere, but agency is not enough: A conceptual analysis of young women’s sexual agency. *Journal of Sex Research,**56*(4–5), 462–474. 10.1080/00224499.2019.1578330 30810374 10.1080/00224499.2019.1578330

[CR5] Berg, S. K., Newins, A. R., & Wilson, L. C. (2022). The effect of social anxiety on the risk of sexual victimization via assertiveness in an ethnically diverse sample. *Violence against Women,**28*(9), 1947–1964. 10.1177/10778012211019044 34160329 10.1177/10778012211019044

[CR6] Blunt-Vinti, H. D., Stokowski, S. E., & Bouza, B. M. (2018). “Your vagina is not supposed to be this scary monster”: Young heterosexual women’s recommendations for improving sexual satisfaction and implications for sexuality education. *American Journal of Sexuality Education,**13*(2), 245–265. 10.1080/15546128.2018.1462279

[CR7] Bond, J. C., & Ford, J. V. (2024). A call for sex-positive epidemiology. *American Journal of Epidemiology,**193*(9), 1205–1210. 10.1093/aje/kwae054 38634632 10.1093/aje/kwae054

[CR8] Byers, E. S., & Rehman, U. S. (2014). Sexual well-being. In D. L. Tolman, L. M. Diamond, J. A. Bauermeister, W. H. George, J. G. Pfaus, & L. M. Ward (Eds.), *APA handbook of sexuality and psychology* (pp. 317–337). American Psychological Association.

[CR9] Cense, M. (2018a). Rethinking sexual agency: Proposing a multicomponent model based on young people’s life stories. *Sex Education,**19*(3), 247–262. 10.1080/14681811.2018.1535968

[CR10] Cense, M. (2018b). Navigating a bumpy road. Developing sexuality education that supports young people’s sexual agency. *Sex Education,**19*(3), 263–276. 10.1080/14681811.2018.1537910

[CR11] Chandra-Mouli, V., Svanemyr, J., Amin, A., Fogstad, H., Say, L., Girard, F., & Temmerman, M. (2015). Twenty years after international conference on population and development: Where are we with adolescent sexual and reproductive health and rights?. *Journal of Adolescent Health,**56*(1), S1–S6. 10.1016/j.jadohealth.2014.09.015 25528975

[CR12] Cherniawsky, S., & Morrison, M. (2022). You should have known better: The social ramifications of victimization-focused sexual assault prevention tips. *Journal of Interpersonal Violence,**37*(1–2), NP125–NP146. 10.1177/0886260520913650 32345092 10.1177/0886260520913650

[CR13] Chmielewski, J. F., Bowman, C. P., & Tolman, D. L. (2020). Pathways to pleasure and protection: Exploring embodiment, desire, and entitlement to pleasure as predictors of black and white young women’s sexual agency. *Psychology of Women Quarterly,**44*(3), 307–322. 10.1177/0361684320917395

[CR14] Crocker, B. C. S., Pit, S. W., Hansen, V., John-Leader, F., & Wright, M. L. (2019). A positive approach to adolescent sexual health promotion: A qualitative evaluation of key stakeholder perceptions of the Australian Australian Positive Adolescent Sexual Health (PASH) Conference. *BMC Public Health,**19*(1), 681. 10.1186/s12889-019-6993-9 31159767 10.1186/s12889-019-6993-9 PMC6547521

[CR15] Delamater, J., & Friedrich, W. N. (2002). Human sexual development. *Journal of Sex Research,**39*, 10–14. 10.1080/00224490209552113 12476250 10.1080/00224490209552113

[CR16] Fine, M. (1988). Sexuality, schooling, and adolescent females: The missing discourse of desire. *Harvard Educational Review,**58*(1), 29–54. 10.17763/haer.58.1.u0468k1v2n2n8242

[CR17] Grose, R. G. (2017). *Critical consciousness and sexual pleasure: Evidence for a sexual empowerment process for heterosexual and sexual minority women* (Doctoral Dissertation). Retrieved from https://escholarship.org/uc/item/3mh413h2

[CR18] Guitelman, J., Mahar, E. A., Mintz, L. B., & Dodd, H. E. (2019). Effectiveness of a bibliotherapy intervention for young adult women’s sexual functioning. *Sexual and Relationship Therapy,**36*(2–3), 198–218. 10.1080/14681994.2019.1660761

[CR19] Haberland, N., & Rogow, D. (2015). Sexuality education: Emerging trends in evidence and practice. *Journal of Adolescent Health,**56*(1), S15–S21. 10.1016/j.jadohealth.2014.08.013 10.1016/j.jadohealth.2014.08.013 25528976

[CR20] Hanbury, A., & Eastham, R. (2015). Keep calm and contracept! Addressing young women’s pleasure in sexual health and contraception consultations. *Sex Education,**16*(3), 255–265. 10.1080/14681811.2015.1093925

[CR21] Hein, S., & Weeland, J. (2019). Randomized controlled trials (RCTs) in clinical and community settings: Challenges, alternatives and supplementary designs. *New Directions for Child and Adolescent Development,**167*, 7–15. 10.1002/cad.20312 10.1002/cad.20312 31509328

[CR22] Hensel, D. J., Von Hippel, C. D., Sandidge, R., Lapage, C. C., Zelin, N. S., & Perkins, R. H. (2021). “OMG, yes!”: Feasibility, acceptability, and preliminary efficacy of an online intervention for female sexual pleasure. *Journal of Sex Research,**59*(3), 269–282. 10.1080/00224499.2021.1912277 34176390 10.1080/00224499.2021.1912277

[CR23] Higgins, J. A., Mullinax, M., Trussell, J., Davidson, J. K., Sr., & Moore, N. B. (2011). Sexual satisfaction and sexual health among university students in the United States. *American Journal of Public Health,**101*(9), 1643–1654. 10.2105/AJPH.2011.300154 21778509 10.2105/AJPH.2011.300154 PMC3154236

[CR24] Impett, E. A., & Tolman, D. L. (2006). Late adolescent girls’ sexual experiences and sexual satisfaction. *Journal of Adolescent Research,**21*(6), 628–646. 10.1177/0743558406293964

[CR25] Jozkowski, K. N. (2013). The influence of consent on college students’ perceptions of the quality of sexual intercourse at last event. *International Journal of Sexual Health,**25*(4), 260–272. 10.1080/19317611.2013.799626

[CR26] Kågesten, A., & Van Reeuwijk, M. (2021). Healthy sexuality development in adolescence: Proposing a competency-based framework to inform programmes and research. *Sexual and Reproductive Health Matters,**29*(1), 104–120. 10.1080/26410397.2021.1996116 10.1080/26410397.2021.1996116 PMC8725766 34937528

[CR27] Katz, J., May, P., Sörensen, S., & DelTosta, J. (2010). Sexual revictimization during women’s first year of college: Self- blame and sexual refusal assertiveness as possible mechanisms. *Journal of Interpersonal Violence,**25*(11), 2113–2126. 10.1177/0886260509354515 20065312 10.1177/0886260509354515

[CR28] Kelley, E. L., Orchowski, L. M., & Gidycz, C. A. (2016). Sexual victimization among college women: Role of sexual assertiveness and resistance variables. *Psychology of Violence,**6*(2), 243–252. 10.1037/a0039407

[CR29] Klein, V., Laan, E., Brunner, F., & Peer, B. (2022). Sexual pleasure matters (especially for women)—data from the German Sexuality and Health Survey (GeSiD). *Sexuality Research and Social Policy,**19*, 1879–1887. 10.1007/s13178-022-00694-y

[CR30] Koepsel, E. R. (2016). The power in pleasure: Practical implementation of pleasure in sex education classrooms. *American Journal of Sexuality Education,**11*(3), 205–265. 10.1080/15546128.2016.1209451

[CR31] Kohler, P. K., Manhart, L. E., & Lafferty, W. E. (2008). Abstinence-only and comprehensive sex education and the initiation of sexual activity and teen pregnancy. *Journal of Adolescent Health,**42*(4), 344–351. 10.1016/j.jadohealth.2007.08.026 10.1016/j.jadohealth.2007.08.026 18346659

[CR32] Laan, E. T. M., Klein, V., van Werner, M. A., Lunsen, R. H. W., & Janssen, E. (2021). In pursuit of pleasure: A biopsychosocial perspective on sexual pleasure and gender. *International Journal of Sexual Health,**33*(4), 516–536. 10.1080/19317611.2021.1965689 38595780 10.1080/19317611.2021.1965689 PMC10903695

[CR33] Lameiras-Fernández, M., Martínez-Román, R., Carrera-Fernández, M. V., & Rodríguez-Castro, Y. (2021). Sex education in the spotlight: What is working? Systematic review. *International Journal of Environmental Research and Public Health,**18*(5), 2555. 10.3390/ijerph18052555 33806507 10.3390/ijerph18052555 PMC7967369

[CR34] Landers, S., & Kapadia, F. (2020). The public health of pleasure: Going beyond disease prevention. *American Journal of Public Health,**110*(2), 140–141. 10.2105/AJPH.2019.305495 31913667 10.2105/AJPH.2019.305495 PMC6951379

[CR35] Levin, D. S., Ward, L. M., & Neilson, E. C. (2012). Formative sexual communications, sexual agency and coercion, and youth sexual health. *Social Service Review,**86*(3), 487–516. 10.1086/667785

[CR36] López-Alvarado, S., Prekatsounaki, S., Van Parys, H., & Enzlin, P. (2022). Sexual assertiveness and its correlates in emerging adults: An exploratory study in Cuenca (Ecuador). *International Journal of Sexual Health,**34*(4), 679–690. 10.1080/19317611.2022.2106527 38596386 10.1080/19317611.2022.2106527 PMC10903662

[CR37] Lorimer, K., DeAmicis, L., Dalrymple, J., Frankis, J., Jackson, L., Lorgelly, P., McMillan, L., & Ross, J. (2019). A rapid review of sexual wellbeing definitions and measures: Should we now include sexual wellbeing freedom? *Journal of Sex Research,**56*(7), 843–853. 10.1080/00224499.2019.1635565 31335208 10.1080/00224499.2019.1635565

[CR38] Mark, K., Corona-Vargas, E., & Cruz, M. (2021). Integrating sexual pleasure for quality & inclusive comprehensive sexuality education. *International Journal for Sexual Health,**33*(4), 555–564. 10.1080/19317611.2021.1921894 10.1080/19317611.2021.1921894 PMC10903684 38595784

[CR39] Mastro, S., & Zimmer-Gembeck, M. J. (2015). Let’s talk openly about sex: Sexual communication, self-esteem and efficacy as correlates of sexual well-being. *European Journal of Developmental Psychology,**12*(5), 579–598. 10.1080/17405629.2015.1054373

[CR40] McGeeney, E., & Kehily, M. J. (2016). Young people and sexual pleasure—where are we now? *Sex Education,**16*(3), 235–239. 10.1080/14681811.2016.1147149

[CR41] Ménard, A. D., & Offman, A. (2009). The interrelationships between sexual self-esteem, sexual assertiveness and sexual satisfaction. *Canadian Journal of Human Sexuality,**18*(1), 35–45.

[CR42] Meston, C. M., & Buss, D. M. (2007). Why humans have sex. *Archives of Sexual Behavior,**36*, 477–507. 10.1007/s10508-007-9175-2 17610060 10.1007/s10508-007-9175-2

[CR43] Miller, J. A., Smith, E. A., Coffman, D., Mathews, C., & Wegner, L. (2016). Forced sexual experiences and sexual situation self-efficacy among South African youth. *Journal of Research on Adolescence,**26*(4), 673–686. 10.1111/jora.12217 28453207 10.1111/jora.12217

[CR44] Mitchell, K. R., Lewis, R., O’Sullivan, R., & Fortenberry, J. D. (2021). What is sexual wellbeing and why does it matter for public health? *The Lancet Public Health,**6*(8), E608–E613. 10.1016/S2468-2667(21)00099-2 34166629 10.1016/S2468-2667(21)00099-2 PMC7616985

[CR45] Oattes, M. K., & Offman, A. (2007). Global self-esteem and sexual self-esteem as predictors of sexual communication in intimate relationships. *Canadian Journal of Human Sexuality,**16*(3–4), 89–100.

[CR46] Oesterle, D. W., Jarnecke, A. M., & Gilmore, A. K. (2022). Sexual re-assault among college women differs based on sexual refusal assertiveness and assertive resistance strategy intentions. *Journal of Interpersonal Violence,**37*(19–20), NP17473–NP17491. 10.1177/08862605211028656 34229531 10.1177/08862605211028656

[CR47] Overeem, A. M., & Van Ditzhuijzen, J. (2022). *De impact van positieve seksuele vorming op seksuele autonomie en seksueel welzijn. Een verkennende review*. Universiteit Utrecht. (Commissioned report for Rutgers, available upon request).

[CR48] Peixoto, M. M., Amarelo-Pires, I., Biscaia, M. S. P., & Machado, P. P. P. (2018). Sexual self-esteem, sexual functioning and sexual satisfaction in Portuguese heterosexual university students. *Psychology & Sexuality,**9*(4), 305–316. 10.1080/19419899.2018.1491413

[CR49] Perkins, T. R., Aleibar, D., Leath, S., & Pittman, J. C. (2023). Black women’s sexual assertiveness and satisfaction: The role of the superwoman schema. *Journal of Black Psychology,**49*(6), 758–784. 10.1177/00957984221147796

[CR50] Phillips, M. A. (2014). *Sexual assertiveness as a predictor of differential vulnerability in sexual victimization between sorority- and non-affiliated college women* (Doctoral dissertation). Retrieved from https://egrove.olemiss.edu/cgi/viewcontent.cgi?article=2518&context=etd

[CR51] Raymond, N. M. (2019). *The effects of a sex positive sexual violence prevention program on college women* (Doctoral dissertation). Retrieved from https://commons.und.edu/theses/4114.

[CR52] Raymond, N. M., & Hutchison, A. N. (2019). A pilot test of the effectiveness of an integrated sex positive education program. *American Journal of Sexuality Education,**14*(3), 315–341. 10.1080/15546128.2019.1584870

[CR53] Rutgers & DUO. (2022). *Relationele en seksuele vorming in het voortgezet onderwijs—Rutgers.* Retrieved from https://rutgers.nl/onderzoeken/relationele-en-seksuele-vorming-in-het-voortgezet-onderwijs/

[CR54] Schry, A. R., & White, S. W. (2013). Sexual assertiveness mediates the effect of social interaction anxiety on sexual victimization risk among college women. *Behavior Therapy,**44*(1), 125–136. 10.1016/j.beth.2012.09.001 23312432 10.1016/j.beth.2012.09.001

[CR55] Scott-Sheldon, L. A. J., Carey, K. B., Cunningham, K., Johnson, B. T., & Carey, M. P. (2015). Alcohol use predicts sexual decision-making: A systematic review and meta-analysis of the experimental literature. *AIDS and Behavior,**20*(S1), 19–39. 10.1007/s10461-015-1108-9 10.1007/s10461-015-1108-9 PMC4683116 26080689

[CR56] Starrs, A., Ezeh, A., Barker, G., Basu, A., Bertrand, J. T., Blum, R. W., & Ashford, L. S. (2018). Accelerate progress—sexual and reproductive health and rights for all: Report of the Guttmacher—Lancet Commission. *The Lancet,**391*(10140), 2642–2692. 10.1016/s0140-6736(18)30293-9 10.1016/S0140-6736(18)30293-9 29753597

[CR57] Sundgren, M., Damiris, I., Stallman, H., Kannis-Dymand, L., Millear, P., Mason, J., & Allen, A. (2022). Investigating psychometric measures of sexual wellbeing: A systematic review. *Sexual and Relationship Therapy,**39*, 1492–1517. 10.1080/14681994.2022.2033967

[CR58] Tolman, D. L., & McClelland, S. I. (2011). Normative sexuality development in adolescence: A decade in review, 2000–2009. *Journal of Research on Adolescence,**21*, 242–255. 10.1111/j.1532-7795.2010.00726.x

[CR59] Townsend, J. M., Jonason, P. K., & Wasserman, T. H. (2020). Associations between motives for casual sex, depression, self-esteem, and sexual victimization. *Archives of Sexual Behavior,**49*, 1189–1197. 10.1007/s10508-019-01482-3 31214905 10.1007/s10508-019-01482-3

[CR60] Tricco, A. C., Lillie, E., Zarin, W., O’Brien, K. K., Colquhoun, H., Levac, D., & Straus, S. E. (2018). PRISMA extension for scoping reviews (PRISMA-ScR): Checklist and explanation. *Annals of Internal Medicine,**169*(7), 467–473. 10.7326/M18-0850 30178033 10.7326/M18-0850

[CR61] Van Bruggen, L. K., Runtz, M. G., & Kadlec, H. (2006). Sexual revictimization: The role of sexual self-esteem and dysfunctional sexual behaviors. *Child Maltreatment,**11*(2), 131–145. 10.1177/1077559505285780 16595847 10.1177/1077559505285780

[CR62] Van Lunsen, H. W., Brauer, M., & Laan, E. (2013). Sex, pleasure and dyspareunia in liberal Northern Europe. In K. S. Hall & C. A. Graham (Eds.), *The cultural context of sexual pleasure and problems: Psychotherapy with diverse clients* (pp. 356–370). Routledge.

[CR63] Vanwesenbeeck, I., Cense, M., Van Reeuwijk, M., & Westeneng, J. (2021). Understanding sexual agency. Implications for sexual health programming. *Sexes,**2*(4), 378–396. 10.3390/sexes2040030

[CR64] Verbeek, M., van de Bongardt, D., Reitz, E., & Deković, M. (2020). A warm nest or “the talk”? Exploring and explaining relations between general and sexuality-specific parenting and adolescent sexual emotions. *Journal of Adolescent Health,**66*(2), 210–216. 10.1016/j.jadohealth.2019.08.015 10.1016/j.jadohealth.2019.08.015 31704106

[CR65] Walker, D. P., Messman-Moore, T. L., & Ward, R. M. (2011). Number of sexual partners and sexual assertiveness predict sexual victimization: Do more partners equal more risk? *Violence and Victims,**26*(6), 774–787. 10.1891/0886-6708.26.6.774 22288095 10.1891/0886-6708.26.6.774

[CR66] Webermann, A. R., Murphy, C. M., Singh, R., & Schacht, R. L. (2022). Preventing relationship abuse among college students: A controlled trial of the skills for healthy adult relationships (SHARE) program. *Journal of Interpersonal Violence,**37*(3–4), NP1860–NP1885. 10.1177/0886260520933033 32564649 10.1177/0886260520933033

[CR67] Wongsomboon, V., Webster, G. D., & Burleson, M. H. (2022). It’s the “why”: Links between (non)autonomous sexual motives, sexual assertiveness, and women’s orgasm in casual sex. *Archives of Sexual Behavior,**51*(1), 621–632. 10.1007/s10508-021-02103-8 34762247 10.1007/s10508-021-02103-8

[CR68] World Health Organization. (2019). *Sexual health*. www.who.int. Retrieved from https://www.who.int/health-topics/sexual-health#tab=tab_1

[CR69] Zaneva, M., Philpott, A., Singh, A., Larsson, G., & Gonsalves, L. (2022). What is the added value of incorporating pleasure in sexual health interventions? A systematic review and meta-analysis. *PLoS ONE,**17*(2), e0261034. 10.1371/journal.pone.0261034 35148319 10.1371/journal.pone.0261034 PMC8836333

[CR70] Zimmer-Gembeck, M. J., See, L., & O’Sullivan, L. (2015). Young women’s satisfaction with sex and romance, and emotional reactions to sex: Associations with sexual entitlement, efficacy, and situational factors. *Emerging Adulthood,**3*(2), 113–122. 10.1177/21676968145480

